# Environmental metal exposure and autism spectrum disorder: spatial analysis of exposure patterns and associated risk among children in Alabama

**DOI:** 10.1007/s11356-026-37566-6

**Published:** 2026-03-06

**Authors:** Oludesola Ogunesan, Cassandra Newsom, Sarah Elizabeth O’Kelley, Kristi Guest, Jeffrey K. Wickliffe

**Affiliations:** 1 Department of Environmental Health Sciences, University of Alabama at Birmingham, Birmingham, AL 35294 USA; 2 Department of Neurobiology, University of Alabama at Birmingham Heersink School of Medicine, Birmingham, AL 35294 USA; 3 Department of Psychology, University of Alabama at Birmingham, Birmingham, AL 35294 USA

**Keywords:** Air pollution, Children’s Environmental Health, Environmental exposure, Air toxic metals, Environmental Risk Assessment, Autism spectrum disorder symptom presentation

## Abstract

Autism spectrum disorder (ASD) is a neurodevelopmental condition with increasing rates of diagnosis and potential links to environmental exposures. Metals are known neurotoxicants that may exacerbate ASD symptom presentation. Our study focused on children aged 3–10 years clinically diagnosed with ASD who have lived in Alabama since birth. Spatial analysis assessed metal distribution and clustering patterns, and Spearman correlation was used to evaluate associations between air toxic metals and ASD symptom presentation. Spatial analysis of airborne metals across Alabama showed considerable spatial variability and hot spots, mostly in central Alabama and surrounding counties. ASD symptom presentation showed moderate positive correlation with cadmium, chromium, and lead, weak positive correlation with manganese, and weak negative correlation with mercury. Proximity and statistical analysis showed 73% of the participants lived within 5 km of an industrial site; those living more than 10 km away had much lower ASD scores, and proximity was a strong predictor of ASD symptoms. Our findings show that children in Alabama may be exposed to multiple air toxic metals at different exposure windows, and industrial emissions and residential proximity to pollution sources are potential environmental contributors to increased ASD symptom presentation.

## Introduction

Autism spectrum disorder (ASD) is a heterogeneous neurodevelopmental disorder with a wide range of neurological impairments, with symptoms usually present before the age of 3, and children with ASD may encounter difficulties in managing their emotions, tone, and facial expressions (CDC 2022; Goodrich et al. 2024; Hodges et al. 2020). ASD is widely recognized as a multifactorial disorder influenced by a combination of genetic, epigenetic, and environmental factors. Although its exact cause remains controversial, research consistently shows that both genetic (inherited traits) and environmental exposures play key roles in ASD development (Shen et al. 2020; Kaur et al. 2021; Ramazani et al. 2024; Zhao et al. 2023; Jafari et al. 2017; Altaf Alabdali1 2014). Environmental exposures, particularly heavy metals such as mercury (Hg), cadmium (Cd), lead (Pb), and aluminum (Al), can cross the blood–brain barrier. This may induce oxidative stress and systemic inflammation during pregnancy and infancy, which may contribute to ASD onset and progression (Kaur et al. 2021; Sulaiman et al. 2020; Adams et al. 2013; Hawari et al. 2020; Mead and Ashwood 2015). Although these metals are naturally occurring, they are also known as universal environmental pollutants produced due to human and industrial activities (Hawari et al. 2020; Zebbiche et al. 2024). According to the World Health Organization, environmental exposure to metals is rising as a result of accelerated industrial activities, product manufacturing, mining, smoking, and emissions from everyday equipment (Kaur et al. 2021; WHO 2024b, 2024a). The acceleration of industrialization has led to heightened levels of pollutants in the environment, thereby increasing exposure to air pollutants, including heavy metals. Populations residing in industrialized or environmentally burdened regions may also face elevated exposure risks (Zota et al. 2016; Zebbiche et al. 2024; Ding et al. 2023; Yenkoyan, Mkhitaryan, and Bjorklund 2024; Sun and Zhu 2019; Lotrecchiano et al. 2022).

Several studies have examined the relationship between air pollution exposure from preconception to pregnancy and childhood, and the increased risk of developing ASD (Bölte et al. 2019; Hertz-Picciotto et al. 2018; von Ehrenstein et al. 2014; Sunyer and Dadvand 2019). These studies have reported increased ASD cases associated with increased environmental pollution, especially heavy metals like mercury, cadmium, and lead (Sulaiman et al. 2020; Talbott et al. 2015; Imbriani et al. 2021; Skogheim et al. 2021; Amadi et al. 2022; Yenkoyan, Mkhitaryan, and Bjorklund 2024).

Metals and their compounds have been associated with an elevated risk of developing and increasing ASD symptoms in the early years of life, a period when the human body is particularly susceptible to environmental toxins (Baj et al. 2021; Zhang et al. 2022; Kalkbrenner et al. 2018). Research suggests that air pollution may be a contributing risk factor for ASD, as estimated annual exposure levels have shown a consistent increase over time (Kalkbrenner et al. 2018; Talbott et al. 2015; O'Sharkey et al. 2025). Air pollution comprises a complex mixture of toxic substances, including various metals, which complicates identifying its biological effects on ASD and the severity of its symptoms (Kalkbrenner et al. 2018; Zhao et al. 2023; Imbriani et al. 2021). Air toxics containing metals enter the body through various routes of exposure, are absorbed into the blood, and travel directly to the brain through the olfactory bulb; the inhalation route presents a higher chance of absorbed metals bypassing the homeostatic control and accumulating in the organs (Baj et al. 2021; Zhang et al. 2021; Ijomone et al. 2020; Jafari et al. 2017; Ventura et al. 2021; Mitra et al. 2022). These metals are known to disrupt critical biological processes and induce epigenetic alterations that alter gene expression without changing the underlying DNA sequence. Such changes can interfere with typical brain development, especially during the sensitive early years of life when neural circuits are rapidly forming, contributing to impaired psychomotor, cognitive, and intellectual development in children (Zebbiche et al. 2024; Saghazadeh and Rezaei 2017; Rezaei et al. 2022; Allesøe et al. 2015; Ding et al. 2023; Kaur et al. 2021). Children are at higher risk of exposure to air pollutants because of their developing organs, including the brain and lungs, and increased outdoor activities (Kitagawa et al. 2022; Zhang et al. 2021; Zierold et al. 2021; McGuinn et al. 2020; Capelo et al. 2022; Ding et al. 2023; Salcedo-Bellido et al. 2024).

The relationship between environmental exposures and health outcomes, such as ASD, can vary depending on the timing of exposure during specific developmental periods, e.g., prenatal, postnatal, and early childhood. Certain time windows of vulnerability to specific exposures may reflect the influence of distinct biological processes occurring during those periods. However, the actual average concentration of air toxics that individuals inhale can differ substantially from the modeled concentrations at specific locations, potentially being either higher or lower (McGuinn et al. 2019; Ali et al. 2019; Weisskopf et al. 2015; Lotrecchiano et al. 2022). Consequently, estimated air toxic concentrations are used as proxies for personal exposure, recognizing that any causal biological mechanisms would occur through actual personal exposure. (Baj et al. 2021; Amadi et al. 2022; Shen et al. 2020; Rashaid et al. 2021; Yu et al. 2024; Zebbiche et al. 2024).

Historically, Alabama has experienced significant air pollution challenges, particularly in industrial hubs, reflecting a longstanding struggle to maintain air quality. Industrialization in Alabama started with the discovery of natural resources, including coal, limestone, and iron ore. This discovery led to iron and steel production and the expansion of railroad networks, establishing the state as a major industrial center in the southern United States (McKiven Jr. 2014; Bond and Rapson 2024; Blau 2022). However, the growth of these sectors has also been linked to environmental air pollution, particularly in communities near industrial facilities compared to other parts of Alabama that are mainly known for agriculture. For example, air quality concerns are significant in the Birmingham area (central Alabama), where pollution levels often surpass federal guidelines (Blau 2022; Maskey and Shinde 2025). Therefore, it is important to assess ASD symptom presentation in relation to multiple airborne metals to which susceptible populations may be exposed in the environment. In this study, we utilize “ASD symptom presentation” to describe a measure usually described as “ASD severity” in existing literature, aligning with the emerging recommendation to use neutral, context-sensitive terminology in ASD research (Singer et al. 2023; Dow and Wang 2025; Dwyer et al. 2025; Keating et al. 2023; Moreira et al. 2025). This study aims to investigate the association between census-block level airborne metals (cadmium, chromium, lead, manganese, and mercury) concentrations estimated using a screening tool and ASD symptom presentation among children in Alabama.

## Materials and methods

### Study area

This cross-sectional study focused on the state of Alabama, located in the southeastern region of the United States of America (USA). According to the Census Bureau (2024), Alabama spans approximately 50,633 square miles and is situated at 32°53′32″ N latitude and 86°42′46″ W longitude on the United States map. The state is bordered by Tennessee to the north, Georgia to the east, Florida to the south, and Mississippi to the west. The most recent U.S. Census (2024) estimates, Alabama’s population is approximately 5.16 million, with 66.5% White, 26.9% Black or African American, 5.3% Hispanic or Latino, and 1.3% Asian. (Census-Bureau 2025; Christy 2022).

### Study design

This study was conducted among children aged 3–10 years, previously diagnosed with ASD, who have resided in Alabama since birth. Race and gender were not used as exclusion criteria. Eligibility criteria were reviewed to confirm that all volunteers met the study requirements prior to enrollment. The children were recruited from February 2023 to August 2025 from the UAB Civitan International Research Center and UAB Civitan-Sparks Clinics. Parents and legal guardians completed a questionnaire providing details such as participants’ socio-demographics, if they have lived in Alabama since conception to birth, if the child was born in Alabama, their current residential address, the duration of their stay at the current residence, and previous addresses within Alabama. Written and signed informed consent was obtained from each participant’s parent or guardian.

### Autism spectrum disorder assessment

Parents or guardians completed release of information forms to allow the study to obtain previously completed ASD assessment data at two UAB clinics. Clinical ASD diagnoses were provided by clinical psychologists conducting comprehensive diagnostic evaluations, including the autism diagnostic observation Schedule, Second Edition (ADOS-2), which is considered a gold standard measure contributing to ASD diagnosis (Greene et al. 2022; Gotham et al. 2012, 2009). Notably, all evaluators were either independent or demonstrated site reliability for research administration of the ADOS-2. ADOS-2 is a standardized and semi-structured observational tool that evaluates ASD symptoms based on communication, social interaction, play, and restricted repetitive behaviors. The ADOS-2 includes five modules that vary in complexity and are selected based on the participant’s age and language development. ASD symptom presentation was measured using the ADOS-2 calibrated severity score (CSS), which is a measure of autism-related symptoms observed during the ADOS-2 relative to similar-aged children with autism at the same language level. CSS is a standardized measure designed to quantify social communication impairments and the presence of restricted and repetitive behaviors (Janvier et al. 2022; Kalb et al. 2022). Higher CSS indicates higher levels of autism spectrum-related symptoms, with a score of 4 or above, suggesting consideration of ASD diagnosis. Specifically, a CSS of 4 and above is associated with a classification of autism spectrum or autism for the ADOS-2, with scores of 5–7 indicating moderate levels and 8–10 indicating high levels of autism spectrum-related symptoms. This study will utilize two main outcome measures to analyze autism diagnosis and phenotype: (a) an ASD diagnosis confirmed during comprehensive clinical evaluation and (b) the level of autism symptom presentation (hereafter referred to as ASD score) evaluated using the CSS, which is widely used in both clinical and research settings (Esler et al. 2015; Kalb et al. 2022; Williams 2022; Greene et al. 2022; Kalkbrenner et al. 2018; Li et al. 2018; Fiore 2020).

### Airborne metal exposure assessment

Participants’ exposures to metals (Cd, Cr, Hg, Mn, Pb) were estimated using the Environmental Protection Agency’s (EPA) 2020 AirToxScreen database, the latest source of modeled hazardous air pollutant (HAP) concentrations. AirToxScreen integrates monitoring data from federal, state, local, and tribal agencies, most notably the State and Local Air Monitoring Stations, which provide daily pollutant measurements (EPA 2024). AirToxScreen builds on its earlier version, the National Air Toxics Assessments (NATA) used from 1999 to 2014. NATA applied the HAPEM model to simulate exposures across pollutants, census tracts, and sources. The 2020 AirToxScreen current framework uses a hybrid modeling system: CMAQ (a 12 km × 12 km chemical transport model) estimates regional pollutant chemistry and transport, while AERMOD (a dispersion model) refines spatial distributions to census block centroids (EPA 2024; Mathieu et al. 2023). This multi-step process yields annual average pollutant concentrations at block-level resolution, balancing chemical accuracy with fine spatial detail. To estimate human exposure, the EPA applies HAPEM8, a screening-level inhalation model that incorporates census data, activity patterns, climate, and indoor/outdoor correlations (Kaufman et al. 2019; EPA 2024). It provides block-level estimates of typical exposures, including ambient and exposure concentrations, and cancer risks. Receptor spacing ranges from 25 m to 10 km depending on census block size (EPA 2024; Kramer et al. 2025; Mathieu et al. 2023).

### Geospatial linkage of exposure data

Airborne metal concentration data from the 2020 AirToxScreen database were spatially linked to participants’ residential locations using Geographic Information Systems (GIS) techniques. Residential addresses were geocoded in ArcGIS to generate precise spatial coordinates. Using spatial joins, multiple datasets, including 2020 AirToxScreen measurements, participant demographics, ASD scores, industry location data, and corresponding census block groups, were systematically integrated. Census tracts were assigned in ArcGIS Pro 3.4 based on the 2021 TIGER/Line files from the United States Census Bureau. This geospatial integration process ensured accurate alignment of airborne metal exposure measurements and ASD scores with participants’ residential environments.

### Statistical analysis

To investigate the association between census-block level airborne metals concentrations (*n* = 128,366) and ASD symptom presentation among children in Alabama, spatial autocorrelation of air toxic metal concentrations was assessed using Moran’s I statistic. Statistical significance was assessed at *p* ≤ 0.05. Moran’s I was computed separately for each metal to quantify the degree of spatial dependence in exposure levels. In addition to Moran’s I, which provides a global measure of spatial autocorrelation, we performed the Getis–Ord Gi* statistic to detect localized patterns of metal concentration clustering at the census block level. This local indicator partitions the study area into smaller spatial units and assigns a Gi* score to each, allowing the identification of statistically significant hot spots and cold spots. Positive Gi* values indicate high-value clusters (hot spots), negative values denote low-value clusters (cold spots), and values near zero suggest spatial randomness. The application of Gi* thus enables a more granular assessment of spatial heterogeneity, complementing the global perspective provided by Moran’s I (Memisoglu Baykal 2025; Khan 2025; Singh et al. 2021).

To test the correlation between the participants’ (*n* = 120) metal concentration measurements and ASD scores, a Spearman correlation test was performed. For each metal, Spearman correlation coefficient (rho) and associated confidence interval (CI) were calculated for each metal.

Participant residential addresses’ proximity to industry locations was assessed using distance-based proximity and buffer-based spatial analyses. Residential mobility was considered by incorporating all known prior and current Alabama addresses (*n* = 205) into our proximity analyses. Euclidean distances from each participant’s residence to the nearest industry were first calculated, providing a continuous measure of distance of industries within 20 km. These continuous measures were then reclassified into threshold categories to generate buffer-based groups at 1 km, 5 km, 10 km, 15 km, and 20 km. Buffer analysis was directly derived from the distance-based proximity measures, enabling both precise statistical modeling of exposure response gradients and categorical classification for mapping, exposure risk assessment, and data interpretation. Distance-based proximity analyses were conducted as screening-level indicators rather than mechanistic exposure models, and the results were interpreted in parallel with modeled exposure estimates. The AirToxScreen concentrations account for atmospheric dispersion and meteorological conditions, including wind patterns, using the CMAQ and AERMOD frameworks (Duan et al. 2022; Shan et al. 2024; Cox 2020; Daniel et al. 2024).

To assess group differences, a one-way analysis of variance (ANOVA) was conducted to test the significance of variations in ASD scores across industry distance, followed by Tukey–Kramer honestly significant difference (HSD) post-hoc tests to account for multiple comparisons and control type I error. Furthermore, we examined the association between industry distance and ASD scores using linear regression analysis. Prior to analysis, normality was assessed using the Shapiro–Wilk test, which indicated significant deviations from normality for both ASD scores and industrial distance (*p* < 0.001). Study participants’ ASD scores (*n* = 120) served as the dependent variable and industry distance as the primary independent variable. A multiple linear regression model was estimated, including age and gender as covariates, to determine whether the relationship between industrial distance and ASD scores remained significant when adjusting for potential confounding. Age is included as a covariate in regression models to account for potential age-related differences in ASD symptom presentation. Model diagnostics were conducted to evaluate assumptions of normality, linearity, and homoscedasticity. Data processing and geospatial analysis was conducted using ArcGIS Pro 3.5.3 for spatial analysis and mapping, and different packages in R statistical software (RStudio 2025.05.1) for statistical modeling.

## Results

### Study population characteristics

Demographics of participants in this study, as seen in Table 1, show that the mean age of the children was 6, with a standard deviation (SD) of 2.1 years (6 ± 2.1). Among the participants, 65.8% are male, and 34.2% are female. 51.7% of the participants have lived in their present residence since birth, while 48.3% have lived in more than one residence since birth. According to the CSS, the ASD score ranged between 4 and 10, with the group mean of 7 indicating a moderate level of ASD symptoms.

**Table 1:** Demographic data and clinical findings of recruited participants

Variables	Statistics or *n* (%)
Age (years)	
3–6	67 (55.8)
7–10	53 (44.2)
Mean age (years) ± SD	6 ± 2.1
Gender	
Male	79 (65.8)
Female	41 (34.2)
Race	
Asian	11 (9.2)
Black/African American	39 (32.5)
Hispanic	9 (7.5)
White	61 (50.8)
ASD CSS	
Mean ASD Score ± SD	7.1 ± 1.3
Residence	
Lived since birth	62 (51.7%)
Lived in one or more	58 (48.3%)

### AirToxScreen metals spatial distribution

The spatial distribution of cadmium, chromium, lead, manganese, and mercury was measured across the state of Alabama. Mean values and corresponding minimum and maximum concentrations were calculated for each metal. The measured concentrations were compared with the United States Environmental Protection Agency (EPA) reference exposure level in Table 2. Comparison with EPA reference exposure level showed that cadmium, chromium, manganese, and mercury maximum concentrations are below the reference exposure level, but manganese maximum concentration exceeds the regulatory threshold.

**Table 2:** Metal concentration at census-block level compared to EPA reference concentration

Metal	Unit	Number of blocks/counties	Mean	Minimum concentration	Maximum concentration	EPA Reference exposure level
Cadmium	µg/m^3^	128,366/67	7.32 × 10^–6^	3.43 × 10^–7^	8.99 × 10^–4^	1 × 10^–2^
Chromium	µg/m^3^	128,366/67	8.77 × 10^–6^	3.21 × 10^–8^	1.68 × 10^–3^	0.1
Lead	µg/m^3^	128,366/67	1.30 × 10^–4^	1.50 × 10^–5^	0.02	0.15
Manganese	µg/m^3^	126,791/67	3.85 × 10^–4^	1.00 × 10^–4^	0.18	5 × 10^–2^
Mercury	µg/m^3^	128,366/67	3.54 × 10^–5^	4.71 × 10^–6^	2.75 × 10^–3^	0.3

*µg/m*^*3*^, micrograms per cubic meter

### Spatial autocorrelation analysis results

Moran’s I spatial autocorrelation analysis indicates strong spatial clustering of metal concentrations across the study area, as shown in Table 3. High Moran’s I values were observed for all metals cadmium (0.98), chromium (0.92), lead (0.86), manganese (0.84), and mercury (0.99), each demonstrating significant spatial autocorrelation (*p* = 0.001). These findings indicate that the distribution of metal concentrations is not random but instead exhibits strong spatial dependence across the state.

**Table 3:** Moran’s I value *p*-values, and z-scores for metals indicate levels of spatial autocorrelation in metal patterns

Metal	Number of blocks/counties	Moran’s I	Z-score	*p*-value
Cadmium	128,366/67	0.98	709.79	0.001
Chromium	128,366/67	0.92	694.81	0.001
Lead	128,366/67	0.86	617.28	0.001
Manganese	126,791/67	0.84	691.36	0.001
Mercury	128,366/67	0.99	707.74	0.001

Furthermore, the high positive Z-scores further support the presence of spatial clustering, with cadmium (Z = 709.79) and mercury (Z = 707.74) showing the strongest clustering patterns. Lead, chromium, and manganese also have high Z-scores, indicating consistent spatial dependence in their spatial distributions.

### Metals hot spot clustering results

**Fig. 1:**
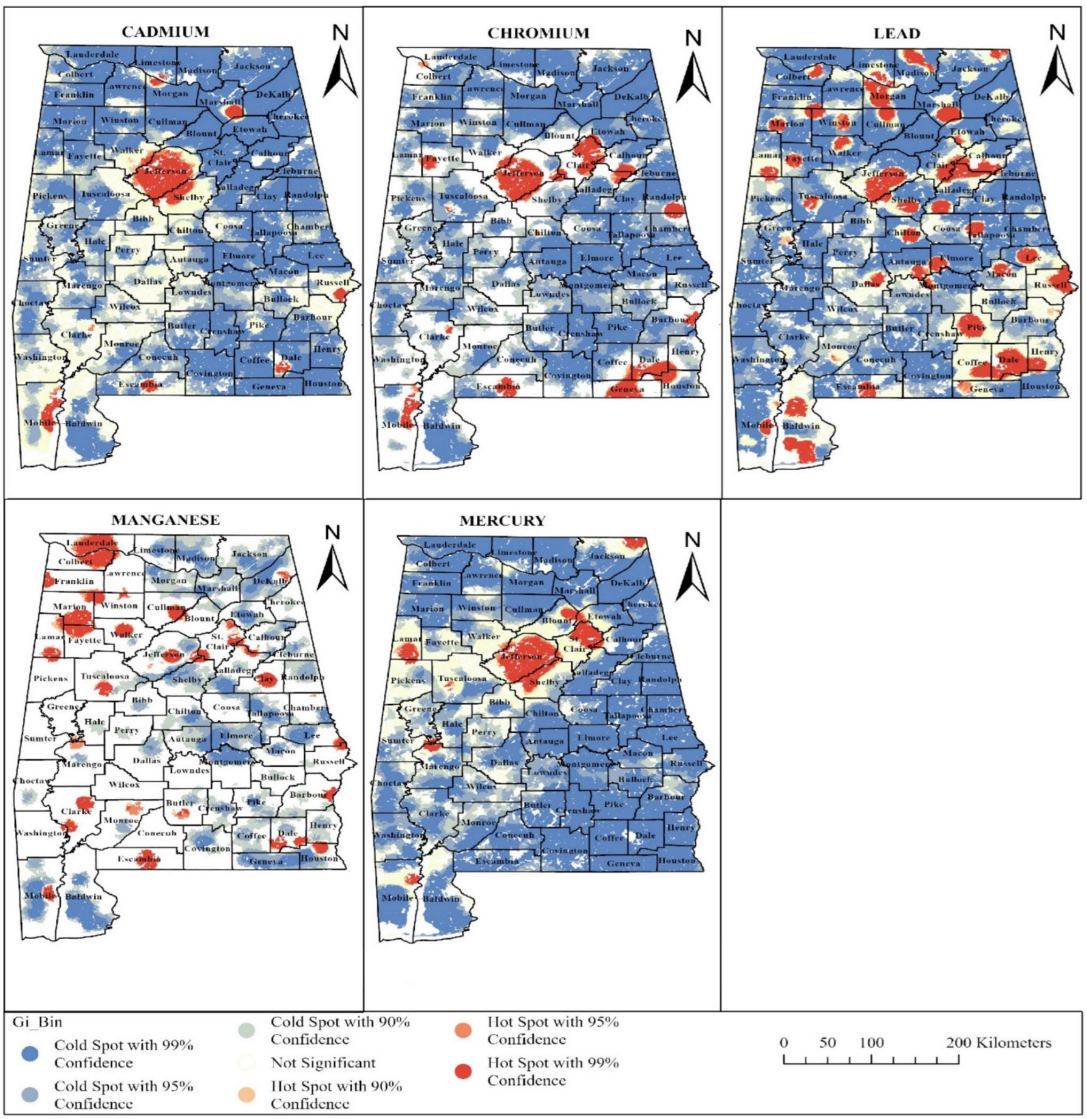
Hot spot maps showing spatial clustering patterns for metal concentrations in Alabama

Getis-Ord Gi* statistic was performed to evaluate the spatial clustering of hot and cold spots for cadmium, manganese, chromium, mercury, and lead across the state of Alabama, as represented in Fig. 1. The analysis was evaluated at 90%, 95%, and 99% confidence intervals, with hot spot areas highlighted at the 99% confidence level, while cold spots highlighted regions of lower-than-expected levels. The analysis shows distinct and complex clustering patterns for each metal, reflecting the influence of both geological and anthropogenic factors. Cadmium's hot spots were concentrated in Jefferson, Shelby, Dale, Russell, Marshall, and Mobile counties, while widespread cold spots were observed across the southern and eastern parts of the state. Chromium showed extensive hot spots across Jefferson, St. Clair, Geneva, Dale, Calhoun, Mobile, and neighboring counties, suggesting strong clustering patterns around industrialized areas, while cold spots dominated much of southern and southeastern Alabama. Lead showed the most widespread hot spot distribution, with clusters in multiple counties along central and northern counties, including Jefferson, Shelby, St. Clair, Cullman, Pike, Dale, Morgan, Marion, Madison, Marshall, Russell, Coffee, Baldwin, and Mobile counties. Cold spots for lead were largely concentrated in the northeastern and southwestern regions, including Lauderdale, Choctaw, Washington, Randolph, and Blount counties. Manganese distribution is more scattered, with multiple hot spots in northwestern counties such as Colbert, Lauderdale, Marion, Fayette, and Franklin, as well as in central and southern counties, including Dale, Clarke, and Escambia counties. Cold spots for manganese were primarily observed in the south and central regions, like Elmore, Baldwin, Tallapoosa, and Montgomery counties. Mercury hot spots were concentrated in Jefferson, St. Clair, Blount, Jackson, Lamar, and surrounding counties, as well as in Marengo and Mobile, whereas cold spots appeared consistently across most other counties.

### Association between EPA AirToxScreen metals concentration and ASD symptom presentation

The Spearman correlation analysis between the metal concentration and participants’ ASD score showed that cadmium, chromium, lead, and manganese were positively associated with increased ASD score. The metals showed a moderate positive correlation with ASD scores with cadmium (rho = 0.62, 95% CI 0.51 to 0.71), lead (rho = 0.57, 95% CI 0.45 to 0.67), and manganese (rho = 0.48, 95% CI 0.34 to 0.60), while chromium (rho = 0.27, 95% CI 0.11 to 0.42) showed a weak positive correlation. Mercury (rho = − 0.24, 95% CI − 0.39 to − 0.08) showed weak negative correlations with ASD scores, indicating a minimal inverse association.

### Association between participants’ residential proximity to industrial sites

The spatial proximity analysis indicated that the distance of participants’ residences to the nearest industry (*n* = 469) varied considerably across the study area. Among the 205 residences (previously lived in and present) analyzed, the mean distance to the closest industry was approximately 4.2 km (SD = 4.2 km), with a median of 2.8 km. Approximately 25% of residences were located within 1.4 km, and 75% were within 5.5 km. The minimum observed distance was 0.28 km, indicating some residents were situated less than 300 m from an industry, while the maximum was 17.5 km, reflecting the outer edge of the study area. When categorized into exposure buffer zones (1 km, 5 km, 10 km, 15 km, and 20 km) from the nearest industry, as demonstrated in Table 4, the industry count within each distance category shows that industrial facilities are more densely situated within the 5 to 10 km range from residential locations, with a decline beyond 10 km. The overall industry count across all distances is 310, with a mean of 77.5, a standard deviation of 52.1, and a median of 56 industries.

**Table 4:** Statistical description at different distance buffers per kilometer (km)

Variable	*N* (%)	Mean ± SD	Confidence interval
**Distance**			
1 km	22	8.86 ± 1.25	(8.31–9.42)
5 km	129	9.31 ± 1.07	(9.03–9.58)
10 km	36	8.71 ± 1.40	(8.12–9.30)
15 km	11	5.50 ± 0.58	(4.58–6.42)
20 km	7	5.80 ± 1.30	(4.18–7.42)
Residence in 20 km vs. Total industry count	205 (66.1%) vs 310		

**Fig. 2:**
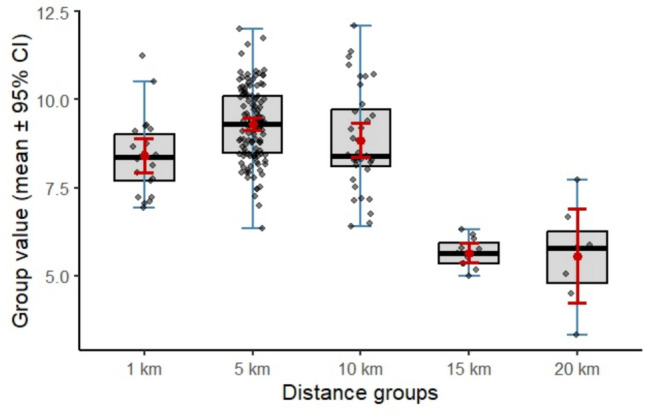
Boxplots of participants’ residential address distance from the nearest industrial site (1–20 km). Boxes show the interquartile range with medians; whiskers indicate range, and points are participants. Red markers and bars denote group means ± 95% CI

**Fig. 3:**
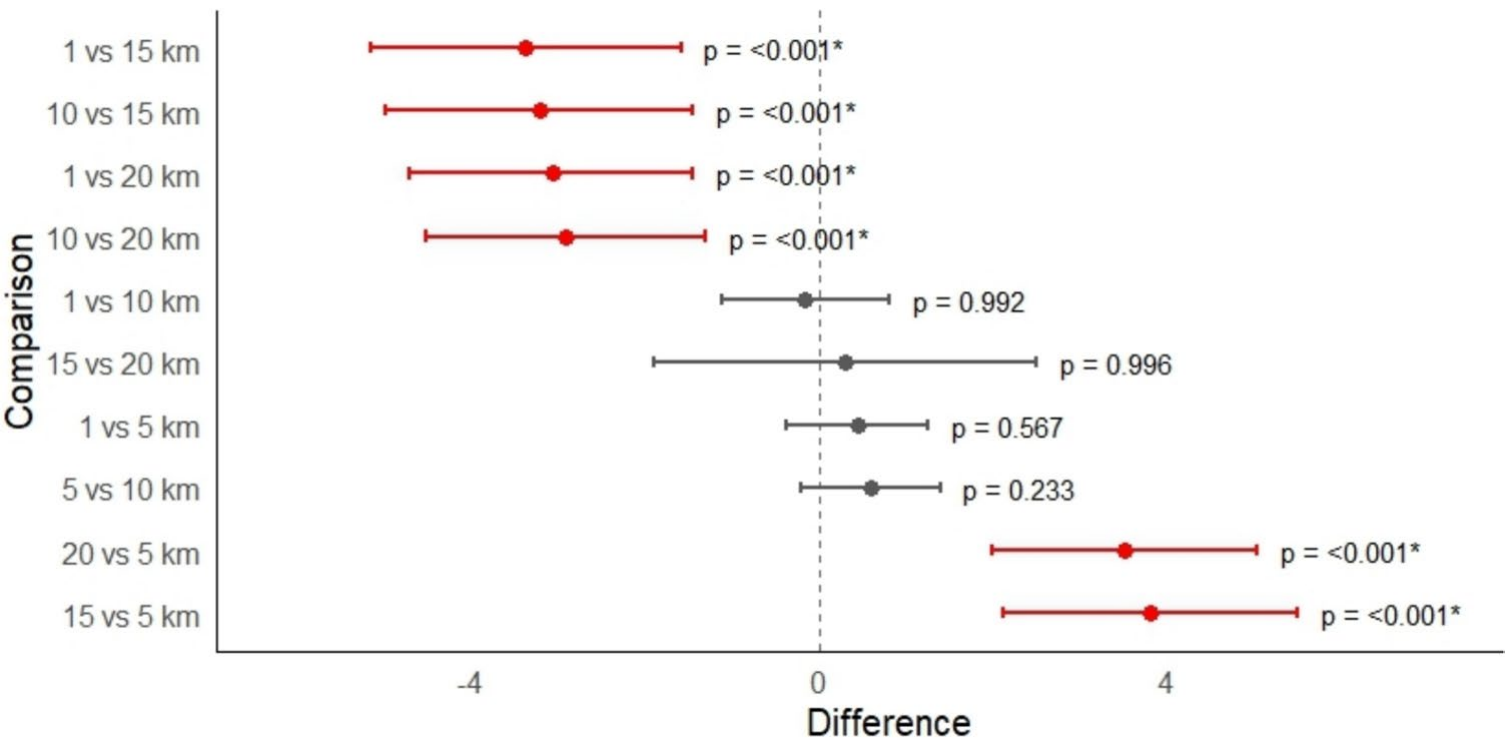
Ordered difference report showing pairwise group comparisons across distance groups. Points indicate mean differences; bars represent 95% CI. Intervals indicate significant difference

The ANOVA analysis in Fig. 2 showed significant differences across distance groups with a p-value < 0.0001. Tukey–Kramer post hoc comparisons showed that the 1 km (8.86 ± 1.25), 5 km (9.31 ± 1.07), and 10 km (8.71 ± 1.40) groups formed a single homogeneous subset (set A), indicating no significant differences among them. In contrast, the 15 km (5.50 ± 0.58) and 20 km (5.80 ± 1.30) groups clustered together (set B), both having significantly lower scores compared to the nearer distance groups. The ordered difference report in Fig. 3 shows that all comparisons between the nearer groups (1–10 km) and the farther groups (15–20 km) were statistically significant (*p* < 0.001), while no significant differences were observed within each cluster. The lack of gradient within 1–10 km likely reflects shared airsheds, non-linear dispersion, and exposure to multiple clustered sources rather than simple distance-based dilution. In the unadjusted linear regression model in Table 5, industry distance was a significant negative predictor of ASD scores (*β* = − 0.25, *p* < 0.001), with higher industry distance associated with lower ASD scores. This model explained 43% of the variance in ASD scores (*R*^2^ = 0.43), suggesting that each additional unit of distance was associated with a 0.43-point reduction in ASD score. In the multiple regression models including age and gender, the negative association between industry distance and ASD scores remained robust (*β* = − 0.25, *p* < 0.001). Gender was also significantly associated with ASD scores (*β* = 0.78, *p* = 0.001), indicating that males received a higher CSS by approximately 0.7 points compared to females, independent of industry distance. Age was not significantly associated with ASD scores (*β* = 0.02, *p* = 0.704), indicating no age-related association. The adjusted model explained 52% of the variance (*R*^2^ = 0.52). Model diagnostics showed that assumptions were largely met, with only minor deviations from normality at the residual tails.

**Table 5:** Regression coefficients predicting ASD symptoms presentation

Predictor	*β*	*t*-value	*p*-value	*R*^2^
Industry distance vs ASD score	−0.25	−9.26	< 0.001	0.43
Industry distance vs gender vs age vs ASD score	−0.25	−9.6	< 0.001	0.52
Gender (female vs. male) vs ASD score	−0.78	−3.52	0.001	
Age vs ASD score	0.02	0.38	0.704	

*β*, regression coefficient; *t*, *p*-value < 0.05 is considered statistically significant; *R*^2^, coefficient of determination

## Discussion

The spatial distribution of cadmium, chromium, lead, manganese, and mercury across Alabama provides valuable insights into potential environmental health risks. Although the mean concentrations for all metals were below the EPA reference exposure level, the range of values demonstrates considerable spatial variability. This suggests that while average exposure levels may not present widespread risks, localized hot spots exist where concentrations are high or near enough to regulatory guidelines. Manganese maximum concentration exceeds the EPA reference thresholds. Although manganese is an essential trace element, chronic exposure has been linked to neurobehavioral and motor deficits (Imbriani et al. 2021; Zhao et al. 2023; Ding et al. 2023; Amadi et al. 2022). Cadmium, chromium, lead, and mercury concentrations were consistently below the EPA reference exposure level across the census blocks. This measurement reflects the effectiveness of longstanding regulatory interventions. Nonetheless, the persistence of detectable levels suggests low-level environmental presence (Martinez-Morata et al. 2023; Zhao et al. 2023; Zhou, Xia, et al. 2025). All these neurotoxic metals have been implicated in contributing to the etiology and progression of neurodevelopmental disorders such as ASD. Several epidemiological studies suggest that early exposure to heavy metals may increase ASD symptoms presentation by interfering with synaptic development and neuronal signaling (Ramazani et al. 2024; Yenkoyan, Mkhitaryan, and Bjorklund 2024; Bhushan 2025; La-Ane et al. 2025; Nehzomi and Shirani 2025; O'Sharkey et al. 2025; da Silva et al. 2025; Zhou et al. 2025a), b). These findings indicate the importance of identifying high-risk areas for more intensive monitoring and potential remediation.

Moran’s I analysis further confirms the presence of significant spatial autocorrelation, indicating that metal concentrations are not randomly distributed but rather exhibit structured spatial dependence. The high Moran’s I values for cadmium (0.9769) and mercury (0.9850) suggest strong localized sources contributing to metal accumulation. The high positive Z-scores further confirm the presence of distinct spatial clusters, reinforcing the importance of environmental factors in determining metal concentrations across the state. These findings align with previous research reporting that residence in areas with high environmental exposure to metals was associated with neurobehavioral outcomes in children, including ASD diagnosis (Capelo et al. 2022; Lotrecchiano et al. 2022). The spatial clustering of cadmium, chromium, mercury, manganese, and lead in Alabama highlights potential environmental exposure gradients relevant to ASD presentation. The consistent hot spots observed in some central Alabama counties, especially Jefferson, Shelby, and their surrounding counties, suggest that long-term industrial emissions and urban activities may have created localized zones of higher exposure. These spreads overlap with areas with elevated population density, raising concerns about the cumulative burden of toxic elements in vulnerable groups, particularly children. Additional hot spots in Mobile County, another industrial and shipping hub, reinforce the role of anthropogenic activities in shaping environmental pollution. We observed cold spots in rural and agriculturally focused areas of the state, which indicate a comparatively lower exposure risk and suggest a lower burden due to reduced industrial and urban activities. Lead hot spots across both northern and southern counties point to legacy or historical pollution sources, while mercury’s concentration in Jefferson, Shelby, and coastal areas suggest industrial or local point sources. Similarly, cadmium and chromium hot spots in urbanized and industrialized areas indicate overlapping exposure risks from multiple metals. Manganese, while an essential nutrient, showed scattered hot spots that could pose risks at elevated levels, especially in combination with other pollutants (McKiven Jr. 2014; Maskey and Shinde 2025; Christy 2022). These patterns suggest that the population, especially children residing in observed hot spot areas, may face higher cumulative exposure burdens, which could contribute to neurodevelopmental outcomes, including ASD. The widespread clustering of lead and mercury, which are known neurotoxic metals and have been linked to ASD in previous studies, further highlights potential exposure pathways relevant to ASD as an outcome. A meta-analysis reported significantly higher concentrations of lead, mercury, cadmium, and arsenic in ASD groups compared to controls (Ding et al. 2023), while other studies demonstrated that children diagnosed with ASD often show elevated concentrations of cadmium, chromium, lead, manganese, and mercury in their hair, toenails, urine, and blood samples (Baj et al. 2021; Zhou, Xia, et al. 2025; Zhao et al. 2023; Zhang et al. 2022; Geier et al. 2012; Ouisselsat et al. 2025; Mogili et al. 2025; Kaur et al. 2025).

This study found both positive and negative correlations between the air toxic metals and ASD scores among children. Our results indicate that cadmium, chromium, lead, and manganese were positively correlated with ASD scores, with cadmium demonstrating the strongest association. The observed correlations were generally positive, although the strength of association was moderate, which may be attributable to the limited sample size. This highlights a potential relationship between exposure to certain heavy metals and increased ASD symptom presentation, underscoring the importance of further investigation into the contributions of environmental exposure to ASD presentation. Our results align with existing literature, suggesting that neurotoxic metals such as lead and cadmium may contribute to adverse neurodevelopmental outcomes, including increased ASD symptoms. While the correlations were not strong, they suggest a more complex interaction between metal exposure and neurodevelopment, potentially influenced by factors such as epigenetic alterations, exposure dose, timing of exposure, or individual susceptibility (Capelo et al. 2022; Amadi et al. 2022; Skogheim et al. 2021; Zebbiche et al. 2024; Xue et al. 2022; Fiore 2020). The weak inverse correlation observed for mercury should be interpreted with caution, as airborne mercury concentrations were relatively low and exhibited limited spatial variability across the study area. Additionally, mercury exposure in children is often related to dietary pathways more than inhalation, suggesting that the negative association may reflect other exposure sources rather than a protective effect (Basu et al. 2018; Sulaiman et al. 2020; Ding et al. 2023; WHO 2024a).

Our ANOVA showed that ASD scores were significantly higher for participants living within 10 km (6.2 miles) of industrial sites compared to those residing 15 and 20 km away. No significant differences were observed among the 1 km, 5 km, and 10 km groups, nor between the 15 km and 20 km groups. This indicates a clear distance-related association, with substantially lower ASD scores beyond 10 km. Our findings indicate that airborne metal concentrations may not exhibit a simple monotonic decay with distance from individual sources. Instead, exposure gradients are shaped by multiple factors, including industrial clustering, overlapping emission plumes, stack characteristics, terrain, and regional background concentrations. Participants located within 10 km are likely to occupy shared airsheds influenced by multiple facilities, thereby reducing exposure contrast across proximal distance categories. The significant differences observed between the near (≤ 10 km) and far (≥ 15 km) groups are consistent with reduced cumulative source influence rather than uniform dilution (EPA 2020; Daniel et al. 2024; Talbott et al. 2015; Shan et al. 2024; Miller et al. 2020). Proximity to industrial sites was a strong predictor of ASD scores, explaining over half of the variance in scores, independent of age and gender. Females showed lower ASD scores than males, while age was not significant. These findings highlight a gradient of exposure potential, with a substantial proportion of participants living or having lived in close proximity (< 5 km) to industries, a range consistent with previous studies identifying this threshold as critical for heightened environmental exposure risks (Daniel et al. 2024; Zota et al. 2016; Zahran et al. 2013; Gong et al. 2014; Capelo et al. 2022). Our result indicates the increasing likelihood of exposure for nearby residents to metals due to their proximity to industrial emissions. This aligns with previous studies' findings that exposure to air pollution, including heavy metals in their present or previous residence, was associated with increased neurodevelopmental risks, reinforcing concerns about the environmental burden on communities residing in industrial areas (Kaufman et al. 2019; Huerta et al. 2023; Capelo et al. 2022).

These findings should be considered in the context of certain limitations. First, the cross-sectional design of the study limits us from establishing causality of the observed associations, and a one-time measurement of airborne metal concentrations limits the ability to assess temporal variations in exposure, potential cumulative effects, and other periodical contributing factors. However, similar previous research, including laboratory animal experiments and longitudinal human studies, reports similar results showing associations and supports the plausibility that prior exposure to heavy metals precedes or potentially contributes to neurodevelopmental disorders in children (Capelo et al. 2022; Lotrecchiano et al. 2022; Kitagawa et al. 2022; Zebbiche et al. 2024; Yu et al. 2024). Second, the use of publicly available EPA 2020 AirToxScreen data may introduce uncertainties in exposure assessment, as air pollution measurements are modeled estimates rather than direct environmental samples. But, given the impracticality of personal biomonitoring for large-scale epidemiological studies, ambient and exposure air concentrations are commonly used as a substitute for assessing individual exposure levels (Mathieu et al. 2023; Kaufman JD 2016). An additional limitation is the unavailability of aluminum in the dataset, which prevented its evaluation in this study. Although prior research has identified aluminum as a neurotoxicant of potential relevance to ASD, its exclusion in this study reflects a data availability constraint rather than a judgment regarding toxicological relevance. Third, while spatial clustering analysis provides valuable insights into block-level, county-level, and regional air pollution exposure patterns, it does not establish causal relationships between air toxic metal exposure, industrial emissions and ASD symptom presentation. Our observed associations may reflect correlated multi-source exposure. Airborne exposure constitutes only one potential pathway of metal exposure; additional sources, including soil, water, diet, and household environments, may also contribute to total body burden. Fourth, the correlation analysis between air toxic metals concentration and ASD scores is limited by our modest sample size, which may affect the generalizability of findings. Our observed spatial patterns may not be generalized, as population density, healthcare access, clinic referral dynamics, and underlying genetic and socioeconomic factors may influence diagnosis and sample representation independent of environmental exposures. Lastly, this study measured participants’ ASD symptom presentation using the ADOS-2 CSS overall scores only, which are relatively high because our participants were volunteers with a diagnosis of ASD recruited from a specialty clinic; this pattern may reflect a selection bias or setting-specific measurements, limiting generalizability to community clinic populations.

Despite the limitations mentioned, our study also includes some strengths. This study demonstrates the intricate relationship between airborne metal concentrations, industrial emissions, and ASD symptom presentation during exposure windows, including prenatal and post-natal exposure. The strong spatial clustering of metals in industrialized regions of Alabama highlights the need for targeted environmental policies and mitigation efforts to reduce emissions from high-risk areas. Furthermore, the correlations between airborne metals and ASD scores suggest that inhalation exposure may play a significant role in systemic metal accumulation, reinforcing the need for biomonitoring in environmental assessment research. Additionally, this is the first study to conduct research investigating the association between ASD scores and environmental exposure in relation to multiple air pollutants that vulnerable populations may be exposed to in the state of Alabama.

Future studies should focus on source assessment models and direct environmental sampling to better identify pollution sources, and updated emission data will further strengthen the interpretation of findings or causal inference. Conducting larger population-based studies in the state with longitudinal exposure assessment would provide more definitive insights into the contributions of metal exposure to ASD symptom presentation across early-life exposure windows, incorporating time-weighted residential histories, age-specific exposure, and multi-pathway exposure assessment. Longitudinal designs better capture critical developmental windows and causal mechanisms. Additionally, a case–control study investigating the contribution of metal exposure to other neurodevelopment disorders among children born and residing in Alabama. Also, identifying sensitive developmental windows and exploring mechanistic pathways will be critical for understanding how exposure to metals influences ASD symptom presentation, from maternal exposure, early stages of life, and childhood. Future investigations may also include children who were evaluated with ADOS-2 who did not receive a diagnosis of ASD to capture the full range of CSS, and access IQ or cognitive assessment data to characterize the sample and investigate the data as an additional factor impacted by and/or impacting ASD symptom presentation.

## Conclusion

This study demonstrates the association between airborne metals concentration and ASD scores, while highlighting significant spatial clustering and exposure disparities across Alabama. Elevated concentrations of metals in industrialized and urbanized counties indicate the impact of localized emissions, particularly around major cities and communities. The strong spatial autocorrelation and hotspot patterns observed further point to structured, non-random distributions of air pollution driven by industrial emissions. The associations between lead, cadmium, and ASD scores highlight the significance of these metals as environmental risk factors. Spatial proximity analyses also show increased exposure risks for residents living within close proximity to industrial facilities, reinforcing concerns about environmental exposure interventions and mitigation. Our findings suggest that identifying and characterizing local sources, alongside policies aimed at reducing emissions, strengthening monitoring networks, and advancing public health interventions, will be critical for mitigating environmental exposure and metal body burden, especially among children in the state of Alabama.

## Data Availability

Data will be available from the corresponding author upon request.

## Declarations

Ethical conduct of research statement

Data was collected under the auspices of IRB approval (IRB-300009712) from children diagnosed with ASD who received research-reliable diagnoses with ADOS-2 at clinics at the University of Alabama at Birmingham.
